# Society Meetings

**Published:** 1897-04

**Authors:** 


### Society Meetings.

The Buffalo Academy of Medicine held meetings during the month of March as follows :

*Section on Medicine.*—Tuesday evening, March 9th, program : Cystitis, by Dr. W. D. Greene ; discussion opened by Drs. Grosvenor and Hartwig. Report of a case of tumor of frontal lobe of brain, with specimen, by Dr. William C. Krauss.

Quarterly meeting of the academy, Tuesday evening, March 16th.

*Section on Pathology* provided the following program : Acute diffuse nephritis, with especial reference to the glomerular changes, by Dr. William T. Councilman, professor of pathology in Harvard Medical School.

*Section on Obstetrics and Gynecology.*—Tuesday evening, March 23d, program: Disorders of menstruation, etiology and pathology, by Dr. Lillian Craig Randall ; Diagnosis and treatment, by Dr. H. D. Ingraham ; The relation of atrophic rhinitis in young women to disorders of menstruation, by Dr. John E. Bacon.

---

The Tri-State Medical Society of Iowa, Illinois and Missouri will be held at St. Louis, April 6, 7 and 8, 1897, under the presidency of Dr. A. H. Cordier, of Kansas City. An interesting program has been issued, which includes clinics, by Drs. Joseph Price, of Philadelphia, James T. Whittaker, of Cincinnati, and Dudley S. Reynolds, of Louisville.

---

The American Medical Association will hold its fiftieth anniversary meeting at Philadelphia, Tuesday, Wednesday, Thursday and Friday, June 1, 2, 3 and 4, 1897, under the presidency of Dr.

Nicholas Senn, of Chicago. The following addresses will be given: Address in surgery, William W. Keen, Philadelphia; address in medicine, Austin Flint, New York; address in state medicine, John B. Hamilton, Chicago. Dr. H. A. Hare, 222 S. 15th street, Philadelphia, is chairman of the committee of arrangements.

---

THE Missouri State Medical Association will hold its next annual meeting at St. Louis, May 18, 19 and 20, 1897. Courtesies will be exchanged between this association and the Illinois State Medical Society, that will hold its annual meeting at East St. Louis on the same date.

---

THE Buffalo Sanatory Club, Dr. Henry R. Hopkins, president, held its regular monthly meeting March 11, 1897, at which an interesting discussion was had upon Buffalo's water supply, and a new site for the city water works pumping station was proposed. The next meeting will be held on Thursday, April 8, 1897.

---

THE Twelfth International Congress will be held at Moscow, August 19 to 26, 1897. Dr. A. Jacobi, 110 W. 34th street, New York, is chairman of the American national committee, to whom inquiries should be addressed.

